# Molecular epidemiology of measles virus in Italy, 2002–2007

**DOI:** 10.1186/1743-422X-9-284

**Published:** 2012-11-23

**Authors:** Fabio Magurano, Claudia Fortuna, Antonella Marchi, Eleonora Benedetti, Paola Bucci, Melissa Baggieri, Loredana Nicoletti

**Affiliations:** 1 Viral Diseases and Attenuated Vaccines Unit National Institute of Health (Istituto Superiore di Sanità, ISS), Rome, Italy

**Keywords:** Measles outbreak, Molecular epidemiology, Genotype, Phylogenetic analysis

## Abstract

**Background:**

The European Regional Office of the World Health Organization (WHO/Europe) developed a strategic approach to halt the indigenous transmission of measles in its 53 Member States by 2015. In view of the goal of measles elimination, it is of great importance to assess the circulation of wild-type measles virus (MV). Genetic analysis is indispensable to understand the epidemiology of measles.

**Methods:**

Urine and saliva samples were collected between May 2002 and December 2007, in order to find the origins and routes of wild type measles virus circulation. RT-PCR was performed on a total of 414 clinical samples of patients from different Italian regions. The results confirmed the genome presence in 199 samples, out of which 179 were sequenced. The sequences were genotyped by comparing the fragment coding for the carboxyl terminus of the nucleoprotein (450 nucleotides) with that one of the WHO reference strains.

**Results:**

From the year 2002 to the year 2007 phylogenetic analysis of measles sequences showed a predominant circulation of the D7 genotype in the Italian territory for the years 2002–2004. This genotype was replaced by D4 and B3 genotypes in the biennium 2006–2007. During the same period C2, A, D5 and D8 genotypes were also detected.

**Conclusions:**

Genetic characterization of wild-type MV provides a means to study the transmission pathways of the virus, and is an essential component of laboratory-based surveillance. Knowledge of currently circulating measles virus genotype in Italy will help in monitoring the success of the measles elimination programme and will contribute to evaluate the effectiveness of future vaccination campaigns.

## Background

Globally, measles morbidity and mortality rates have been dramatically reduced since 1963, as a result of the availability of a safe and effective vaccine and the implementation of enhanced vaccination strategies [1-3]. Interruption of indigenous transmission of measles virus (MV) (Paramyxoviridae; Morbillivirus) has been reported for several countries [4]. Nonetheless, measles remains a leading cause of childhood mortality worldwide, with an estimated 164,000 measles deaths in 2008 (a 78% reduction compared to mortality rate in 2000), most of which took place in developing countries, primarily because of underutilization of the vaccine [5,6]. Furthermore, large outbreaks continue to occur in countries with high vaccination coverage, after importation of the virus from endemic regions [7]. WHO measles elimination plan in the European Region (EUR) is targeted by 2015 [1]; thus, progress in measles control in this region is fundamental for the success of the measles elimination programme [8]. In 2002–2003, Italy experienced a large measles outbreak affecting mostly the Southern regions, with an estimated national incidence of 738/100,000 in 2002 and 544/100,000 in 2003, in children below 15 years of age, corresponding to over 100,000 estimated cases in this age group [9-11]. The outbreak was due to poor measles vaccination coverage. In fact, in 2003 the Italian national rate of children vaccinated with one dose of measles-containing vaccine by 24 months of age was 77%, being significantly lower in Southern regions compared to Central and Northern areas [12,13].

Measles is a statutory and notifiable disease in Italy. During a six-year period (2001–2006) an average of approximately 5,400 cases was reported annually, with a range from 18,020 cases in 2002, to 215 cases in 2005. According to the National Elimination Plan, sensitivity, specificity, and timeliness of case reporting had to be improved. Therefore, in April 2007 an enhanced surveillance system was established [14], and a National Reference Laboratory (NRL) was established at the Istituto Superiore di Sanità (ISS) in order to support cases ascertainment, confirm outbreaks/cases and determine the MV genotypes.

Molecular epidemiology, i. e. genetic characterization of wild-type MVs combined with standard epidemiological methods, is an essential component of the laboratory-based surveillance. It is performed throughout the world by the WHO Measles and Rubella Laboratory Network, which serves 166 countries in all WHO regions. Virological surveillance has helped to document the interruption of transmission of endemic measles in some regions. It includes epidemiological investigation and laboratory confirmation of all sporadic illnesses clinically consistent with measles. Moreover, laboratory-based surveillance for measles and rubella, including genetic characterization of wild-type viruses, permits to illustrate the progress towards measles elimination by differentiating viruses between indigenous and imported. Molecular characterization of measles viruses provides a valuable tool to measure the effectiveness of measles control programmes, and virological surveillance needs to be expanded throughout the world, and conducted during all phases of measles control.

Genetic characteristics of representative wild-type MVs, identified in Italy between 2003 and 2007, were analyzed in this study.

## Results

**Figure 1:**
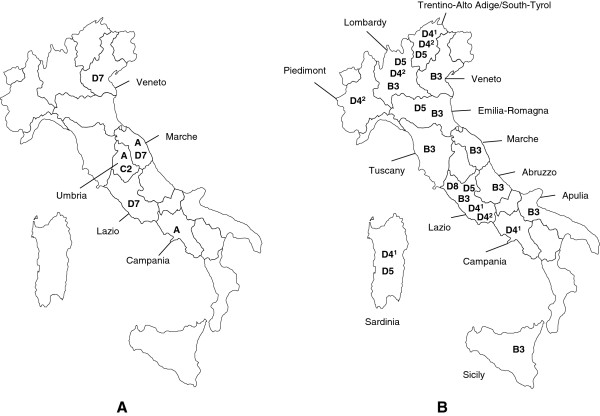
Geographical distribution of measles virus (MV) genotypes isolated in Italy, between 2002 and 2004 (A), and between 2006 and 2007 (B).

Urine and saliva samples were collected between May 2002 and December 2007 (Figure 1) in order to find origins and routes of MV wild-type circulation. RT-PCR (reverse transcriptase polymerase chain reaction) was performed on a total of 414 clinical samples, 399 urine and 15 saliva respectively, collected from different Italian regions and coming from 414 patients. The results confirmed the presence of the genome in 199 samples, out of which 179 were sequenced (Table 1). The sequences were genotyped by comparing the fragment coding for the carboxyl terminus of the nucleoprotein (450 nucleotides) with the one of the WHO reference strains. A representative set of sequences is listed in Table 2.

**Table 1:** **Measles cases tested at the Italian National Reference Laboratory** (**ISS**) **during the period 2002**–**2007**, **and related genotypes**

	**N of cases**			**Genotypes**						
**Year**	**Tested samples**	**RT**-**PCR positive**	**Sequenced**	**A**	**B3**	**C2**	**D4**	**D5**	**D7**	**D8**
**2002**	38	23	23	2	-	-	-	-	21	-
**2003**	19	7	7	-	-	-	-	-	7	-
**2004**	21	4	4	1	-	3	-	-	-	-
**2005**	no data	no data	no data	-	-	-	-	-	-	-
**2006**	127	93	76	-	16	-	60	-	-	-
**2007**	209	72	69	-	35	-	29	4	-	1
**TOT**	414	199	179	3	51	3	89	4	28	1

**Table 2:** List of the representative sequences analyzed between 2002 and 2007

**Year**	**Name of strain**^**1**^	**N of sequences**	**N of weeks**^**2**^	**Genotype**	**GenBank accession number**	**Total**
2002	MVs/Roma.ITA/20.02/1	1	1	D7	JQ783051	23
	MVs/Roma.ITA/20.02/2	3	6	D7	JQ783052	
	MVs/Ancona.ITA/26.02	1	1	D7	JQ783050	
	MVs/Caserta.ITA/27.02/1	5	1	D7	JQ783049	
	MVs/Caserta.ITA/27.02/2	1	1	A	JQ783038	
	MVs/Ascoli.ITA/27.02/1	8	4	D7	JQ783048	
	MVs/Ascoli.ITA/30.02/5	1	1	A	JQ783039	
	MVs/Ancona.ITA/45.02	3	1	D7	JQ783047	
2003	MVs/CastelfrancoVeneto.ITA/09.03	1	1	D7	JQ783040	7
	MVs/Roma.ITA/09.03	1	1	D7	JQ783041	
	MVs/Avezzano.ITA/14.03	1	1	D7	JQ783042	
	MVs/Roma.ITA/15.03	1	1	D7	JQ783043	
	MVs/Pistoia.ITA/23.03	1	1	D7	JQ783044	
	MVs/Pordenone.ITA/19.03/1	1	1	D7	JQ783045	
	MVs/Pordenone.ITA/19.03/2	1	1	D7	JQ783046	
2004	MVs/Todi.ITA/28.04/1	3	1	C2	EF490999.1	4
	MVs/Todi.ITA/28.04/4	1	1	A	EF469771.1	
2006	MVs/Merano.ITA/33.06	2	1	D4^1^	AM849094.1	75
	MVs/Roma.ITA/33.06/1	38	18	D4^1^	EF533887.1	
	MVs/Latina.ITA/35.06	7	7	D4^1^	JQ783032	
	MVs/Alghero.ITA/36.06	3	2	D4^1^	JQ783033	
	MVs/Roma.ITA/36.06	6	4	D4^1^	JQ783034	
	MVs/Roma.ITA/37.06/7	1	1	D4^1^	JQ783035	
	MVs/Pescara.ITA/39.06	1	1	D4^1^	JQ783036	
	MVs/Roma.ITA/43.06/2	9	10	B3.1	EF533886.1	
	MVs/Roma.ITA/45.06/2	1	1	D4^1^	JQ783037	
	MVs/Pomezia.ITA/48.06/1	2	4	B3.1	JQ783028	
	MVs/Albano.ITA/48.06	3	2	B3.1	JQ783029	
	MVs/Aprilia.ITA/49.06	1	1	B3.1	JQ783030	
	MVs/Latina.ITA/50.06	1	1	B3.1	JQ783031	
2007	MVs/Roma.ITA/01.07	8	14	B3.1	JQ783008	69
	MVs/Roma.ITA/02.07	1	1	B3.1	JQ783009	
	MVs/Barletta.ITA/03.07	1	1	B3.1	JQ783010	
	MVs/CapoD'Orlando.ITA/04.07	1	1	B3.1	JQ783011	
	MVs/Latina.ITA/07.07	1	1	B3.1	JQ783012	
	MVs/Salerno.ITA/07.07	1	1	D4^1^	JQ782998	
	MVs/Merano.ITA/07.07	1	1	D4^1^	JQ782999	
	MVs/Milano.ITA/09.07	1	1	D4^2^	JQ783007	
	MVs/Roma.ITA/10.07/1	5	5	B3.1	JQ783013	
	MVs/Roma.ITA/10.07/3	1	1	B3.1	JQ783014	
	MVs/Grosseto.ITA/11.07	1	1	B3.1	JQ783015	
	MVs/Parma.ITA/11.07	7	9	B3.1	JQ783016	
	MVs/Milano.ITA/11.07	1	1	B3.1	JQ783017	
	MVs/Viterbo.ITA/11.07	1	1	B3.1	JQ783018	
	MVs/Roma.ITA/12.07	1	1	D8	JQ783053	
	MVs/Roma.ITA/13.07	3	1	D4^2^	JQ783006	
	MVs/Ancona.ITA/13.07	3	4	B3.1	JQ783019	
	MVs/Viterbo.ITA/13.07	1	1	B3.1	JQ783020	
	MVs/Ferrara.ITA/15.07	1	1	B3.1	JQ783021	
	MVs/Bari.ITA/21.07	1	1	B3.1	JQ783022	
	MVs/Padova.ITA/23.07	1	1	B3.1	JQ783023	
	MVs/Bolzano.ITA/38.07	1	1	D5	JQ783027	
	MVs/Lodi.ITA/38.07	1	1	D5	JQ783025	
	MVs/Roma.ITA/38.07	1	1	D5	JQ783024	
	MVs/Bologna.ITA/39.07	1	1	D5	JQ783026	
	MVs/Cuneo.ITA/41.07	12	7	D4^2^	JQ783005	
	MVs/Milano.ITA/41.07/2	1	1	D4^2^	JQ783004	
	MVs/Cuneo.ITA/46.07	7	1	D4^2^	JQ783003	
	MVs/Trento.ITA/47.07/1	1	1	D4^2^	JQ783002	
	MVs/Trento.ITA/47.07/2	1	1	D4^2^	JQ783001	
	MVs/Torino.ITA/51.07/1	1	1	D4^2^	JQ783000	

^1^: The nomenclature indicates the geographical origin and the year in which each virus was first identified. Identical sequences collected in the same place were grouped, and a single representative sequence was submitted in GenBank.

^2^: Period of circulation of each strain from its first isolation.

**Figure 2:**
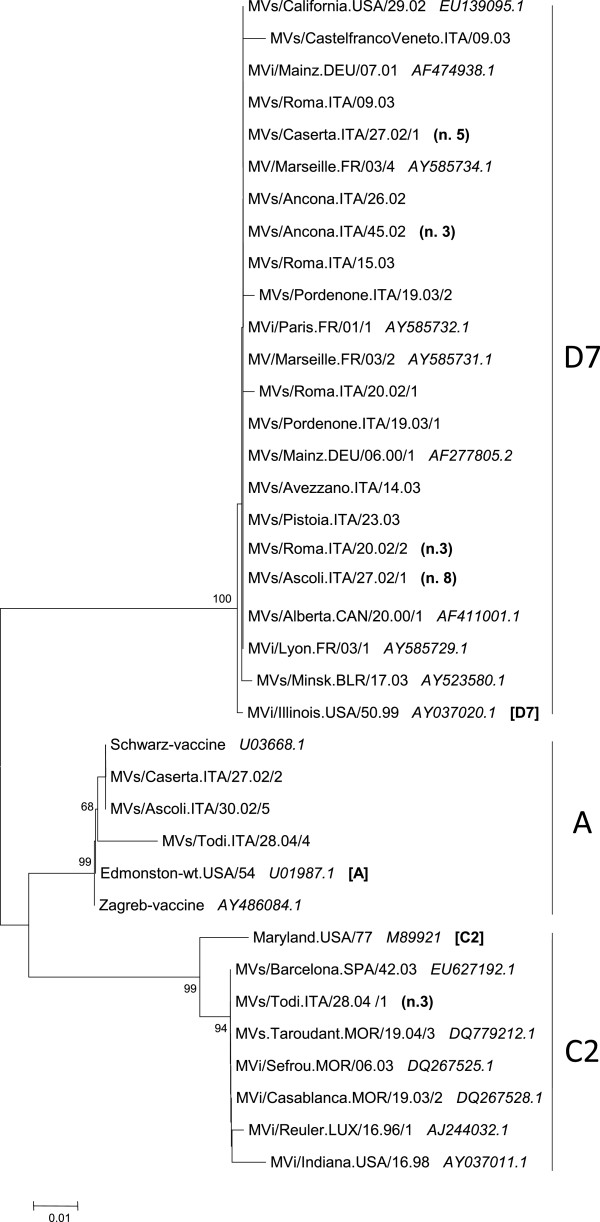
**Phylogenetic analysis according to the partial sequence of the N gene of measles virus (MV)strains identified in Italy,from 2002 to 2004.** Numbers in parenthesis indicate the number of additional identical strains from the same region. Significant bootstrap values (>80%) are indicated.

The results of genetic analysis indicated that 21 out of 23 specimens sequenced in 2002, either before or during the peak of the outbreak, belonged to genotype D7 and 2 to genotype A (Figure 2). All the 21 sequences classified as genotype D7 were closely related to each other, showing overall only a single nucleotide difference. This fact supports the hypothesis of a common origin of the epidemic. Furthermore, these strains showed a 100% identity with those isolated in France in 2001 and 2003 (MVi/Paris.FR/01/1, MVi/Lyon.FR/03/1), in Germany in 2000 and 2001 (MVs/Mainz.DEU/06.00/1, MVi/Mainz.DEU/07.01), in Canada in 2000 (MVs/Alberta.CAN/20.00/1), and in Belarus in 2003 (MVs/Minsk.BLR/17.03), and were 99% identical to the MV D7 reference strain MVi/Illinois.USA/50.99.

During 2003, 7 specimens were sequenced: all of them belonged to genotype D7. All these sequences were closely related to each other, showing 99-100% identity with the D7 strains circulating in Italy in 2002, and with the strains circulating in Europe during the years 2000–2003 (Figure 2).

During 2004, 4 specimens were sequenced: 3 of them belonged to the genotype C2 and 1 to genotype A. MVs strains belonging to the genotypes C2 circulated in Germany in 1992, in Spain during 1992–1993, and in Great Britain during 1992–1995, suggesting a wide distribution of this genotype throughout Europe. More recently, genotype C2 has been detected in Luxembourg as well as in Germany, the Czech Republic and Denmark; it was responsible for the epidemic that occurred in Morocco in 2003. The Italian C2 strain differed for a single nucleotide from that one isolated in Luxembourg during 1996–1997 [15], as well as from two strains imported into the USA in 1997–1998 [16], while it differed only for one nucleotide from the C2 Moroccan strain (Figure 2). No epidemiological links are available for these strains. The Italian C2 strains showed a sequence identical to the Moroccan and Spain strains circulating in 2003 (MVi/Casablanca.MOR/19.03/2).

All the 3 strains belonging to the genotype A, circulating in 2002 and 2004, revealed a close relationship with the vaccine strains: the strains isolated in 2002 showed identical sequences and the other A strain showed the 90% of identity with the vaccine sequence (Figure 2). The strains isolated in the period 2002–2004 came from Campania, Lazio, Umbria Marche and Veneto regions (Figure 1).

No laboratory data are available for the year 2005 because of the interruption of the surveillance activity that restarted in the year 2006.

**Figure 3:**
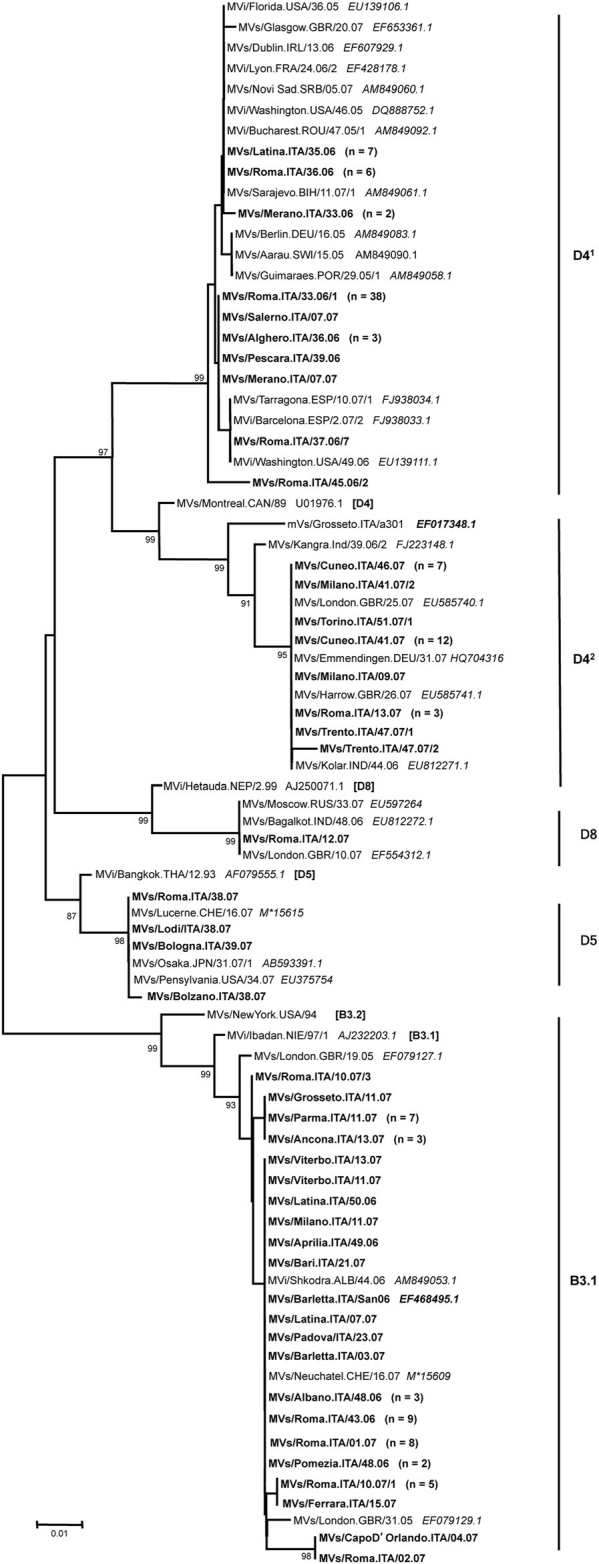
**Phylogenetic analysis according to the partial sequence of the N gene of measles virus (MV) strains identified in Italy, in the biennium 2006–2007.** Numbers in parenthesis indicate the number of additional identical strains from the same region. Significant bootstrap values (>80%) are indicated. M*: MeaNS id number.

During 2006, 75 specimens were sequenced. The phylogenetic analysis showed that 59 sequences out of 76 belonged to genotype D4 and 16 to the genotype B3 (Figure 3).

In 2007, 69 sequences were analyzed; 29 of them belonged to genotype D4, 35 to genotype B3, 4 to genotype D5 and 1 to genotype D8.

Therefore, the majority of MVs circulating between 2006 and 2007 in Central and Northern Italy belonged to genotype D4 (n= 88), and appeared to be grouped into two different clusters: D4^1^ (n= 61) and D4^2^ (n= 27), respectively (Figure 3).

The 73% (44 out of 60) of the D4^1^ sequences were 100% identical to each other; 22% (13 out of 60) differed from the latter for a single nucleotide; 3% (2 out of 60) showed overall a difference of three nucleotides, and 1.6% of five nucleotides (1 out of 60).

BLAST analysis in MeaNS database (Measles Nucleotide Surveillance; http://www.who-measles.org) showed that the D4^1^ cluster sequences were for the 99% identical to the strains that circulated in Europe and then imported into the USA in the years 2006 and 2007 [17]. The strains belonging to this cluster were first isolated in February 2006 and circulated until February 2007 in Trentino Alto Adige, Lazio [18] and Sardinia regions.

The measles viruses belonging to the cluster D4^2^ were first isolated in February 2006 and circulated throughout the entire year in Trentino Alto Adige, Lombardy, Piedmont, Lazio and Sardinia regions (Figure 1). These sequences showed a maximum difference of 2 nucleotides (≤0.5%), difference that remained unchanged when the strains MVi/Kolar.IND/03.07/1 and MVs/London.GBR/25.07 were added to the alignment, while it showed an increase with the addition of the strain MVs/Grosseto.ITA/a301 [19].

Instead, the 51 B3 strains, isolated during the biennium 2006–2007, belonged to the subgroup B3.1 (Figure 3) [20]. The genetic diversity of all these B3.1 circulating strains was limited to a maximum difference of 4 nucleotides (≤0.99%). In addition, the genetic analysis revealed that some of these Italian cases were also identical to a case from Albania in 2006 (MVi/Shkodra.ALB/44.06), and to a case from Great Britain in 2005 (MVs/London.GBR/31.05). The sequences belonging to B3.1 genotype were first isolated in October 2006 and the relevant strains circulated until June 2007 in Veneto, Lombardy, Emilia Romagna, Tuscany, Marche, Lazio, Abruzzo, Apulia and Sicily regions.

## Discussion

This study stands so far as the first description of the molecular epidemiology of MV in Italy.

Before this study the information about strains circulating in Italy derived from some cases occurred in Italy and imported into the U.S.A., Great Britain and Luxembourg [21,22]. The genotypes described were D6 (1996–97) and D8 (1999).

In our study we found that 28 (82%) out of the 34 MV sequences detected from clinical specimens during 2002–2004 belonged to genotype D7. Three (9%) were placed in genotype C2 and three (9%) were placed in genotype A (Figure 2).

The results of our analysis indicate that genotype D7 was the cause of the epidemic that occurred in 2002, and probably of the one occurred in 2003, with 18,020 and 11,978 measles confirmed cases, respectively. Therefore, genotype D7 was the endemic MV in the years 2002 and 2003. The sequence analysis of the Italian cases showed that all the strains identified were strongly related to each other, and closely related to the European strains.

Genotype D7 circulated in Great Britain and in Australia during the 1980s [23,24]. Chains of transmission of this genotype have been associated, through epidemiological investigations, to Sweden and other European countries [25]. In the early 2000s, this genotype replaced the genotypes C2 and D6, becoming the most commonly isolated genotype in Germany and France [26,27]. This shift was demonstrated in Germany during 2000–2001 [28]. Genotypes shift takes place in those countries where the interruption of endemic transmission occurs only for short periods, due to measles sub-optimal control programmes. Indeed, failure in maintaining high levels of population immunity results in the accumulation of susceptible individuals, thus creating favourable conditions for a rapid transmission of a newly introduced genotype.

The comparison of the Italian A genotype MVs with those deposited in Means database revealed a close relationship with the Schwarz Italian vaccine strains, as well as with the wild-type genotype A strain (Figure 2). Unfortunately, no information about the vaccination status of these patients was available. However, the close genetic relationship with the vaccine strains indicates that these cases should be caused by vaccine virus.

Phylogenetic analysis for the sequences of the Italian cases in the years 2006–2007 revealed a co-circulation of D4 and B3 genotypes. In addition, we identified an outbreak caused by genotype D5, and only a single case belonging to D8 genotype in 2007 (Table 2; Figure 3).

Eighty-nine (61%) out of the 145 MV strains detected in clinical specimens, in the years 2006–2007, belonged to genotype D4. This genotype was endemic on the Indian subcontinent, as well as in East and South Africa [25,29]. Genotype D4 has been repeatedly identified in the WHO Eastern Mediterranean Region [30], as well as in outbreaks and sporadic cases in several European countries, including Germany, Turkey, Spain, Great Britain, Croatia and Russia [17,31,32].

Italian D4 strains grouped into two different clusters of common origin (Figure 3). The strains belonging to cluster D4^1^ were isolated from August 2006 to February 2007, and were closely related to the strains circulating in Europe and USA from late 2005 to early 2007. According to the analysis made by Kremer et al. [17] these strains belonged to a group described as ‘European group 1’. Moreover, this group was responsible for a large outbreak in Romania, which included >8,000 cases and lasted from December 2004 until early 2007 [17].

D4 Italian strains belonging to cluster D4^2^ were isolated from November 2006 to December 2007, and were closely related to the Grosseto strain [19] and to the strains circulating in India, Great Britain and Germany in the same period. D4^2^ was responsible for the outbreak occurred in Piedmont in 2007 having been imported from the United Kingdom [14] and differed from the European groups that circulated in the biennium 2005–2006 [17].

In 2007 the strain D5 caused a small outbreak in Italy, and the phylogenetic analysis suggests that it was probably imported from Japan. In fact, despite unavailability of epidemiological links, this strain caused an outbreak in 2007 in Hokkaido district (Japan) [33].

In 2007 we found a single case belonging to the D8 genotype (Figure 3), whose strain has been isolated in Russian [34] and was endemic in India in the same period [35] but the source of the virus remained unknown.

From October 2006 to December 2007 the circulation of B3 strain, the same strain that caused the Apulia outbreak in 2006 [36], was observed with the first case identified. The strain was identified for the first time in the Lazio Italian region. This could suggest the place where the B3 outbreak started, but no epidemiological data supported this hypothesis.

Fifty-one (35%) of the MV strains detected in clinical specimens in the years 2006–2007 belonged to genotype B3. All these strains belonged to genotype B3.1 and were closely related to the strains isolated in Albania, Switzerland and circulating in Great Britain in the same period, suggesting a possible route of introduction by the latter (Figure 3) [14] although the origin of B3 outbreak was not known.

In summary, according to our analyses we can assert that D7 strain was endemic in Italy from 2002 to 2004. In 2006 it was replaced by D4 and B3 strains that circulated also in 2007.

In conclusion, a continuous improvement of laboratory surveillance is needed, particularly of the molecular laboratory. This is significantly important in countries where the measles elimination phase, such as Italy, is taking place and increasing efforts to obtain appropriate specimens from each chain of transmission are necessary.

## Materials and methods

### Measles case definitions

Measles cases were clinically defined i.e., generalized rash, lasting at least 3 days, accompanied by fever > 38°C, and cough, coryza or conjunctivitis.

Laboratory confirmation was attained by the determination of measles specific IgM antibodies through enzyme immunoassay, and/or by the detection of measles virus genome in saliva or urine by RT and hemi-nested PCR.

### Specimens

Oral-fluid specimens were obtained by salivary swabs (Salivette, Sarstedt Company). Saliva was collected after centrifugation at 500 g for 10 minutes, and then stored until tested.

Urine samples were collected within 7 days from rash onset. Urine sediment was obtained after centrifugation at 500 g for 10 minutes, washed two times in sterile PBS, and resuspended in a final volume of 0.5-1 ml. Sediments were stored at −80°C. The tests on urine and salivary samples were performed under the indications of the “National Plan of Elimination of Measles and Congenital Rubella”. The Plan has been elaborated by the Ministry of Health according with the WHO indications, and approved by all Regional Health Authorities. Our laboratory belongs to the WHO European Regional Network of National Measles and Rubella Reference Laboratories. Goal for this network is the measles elimination in Europe in 2015. Before collecting samples, subjects, or their parents in case of children, had to sign the “informed consensus”. No formal approval from an ethics committee has been required; however the study complies with the Helsinki declaration.

### RT-PCR amplification and sequencing

Total RNA was extracted using QIAmp Viral RNA Mini Kit (Qiagen) for saliva samples and RNeasy mini Kit (Qiagen) for urine samples, as per manufacturer protocols. Nucleic acid was tested by RT-PCR using a hemi-nested protocol [24] directed to a highly conserved part of the MV RNA, which is located on the N gene. Kit SuperScript One-Step RT-PCR kit with Platinum Taq (Invitrogen) was used for RT-PCR reaction.

### Nucleotide Sequence Analysis

Both strands of amplified products were sequenced by Macrogen Inc. (Seoul, Korea), using MVF2 and MVB1 primers.

Genomic sequences of reference strains used for genetic analysis of wild-type measles viruses were obtained from database by accession number [37,38]. Nucleotide sequences were aligned with the CLUSTAL W (BioEdit) software [39]. Phylogenetic trees were constructed using the nucleotide Kimura-2 parameter and the neighbour-joining method. Bootstrap analyses were performed through 1,000 resampling of the data sets. The neighbour-joining method [40] was implemented by using MEGA-5 [41].

The sequences had been submitted to GenBank, and the corresponding accession numbers are given in Table 1.

## Abbreviations

WHO: World Health Organization; MV: Measles virus; RT-PCR: Reverse transcriptase polymerase chain reaction; EUR: European Region; ISS: Istituto Superiore di Sanità; NRL: National Reference Laboratory; N: Nucleoprotein.

## Competing interests

The authors declare that they have no competing interests.

## Authors' contributions

FM carried out most of the studies and drafted the manuscript. LN designed the study and organized the coordination. CF, AM, EB, PB collected specimens, performed RT-PCR and viral identification. MB, AM, CF performed parts of the studies and provided consultation and editing of the manuscript. All authors read and approved the final manuscript.
